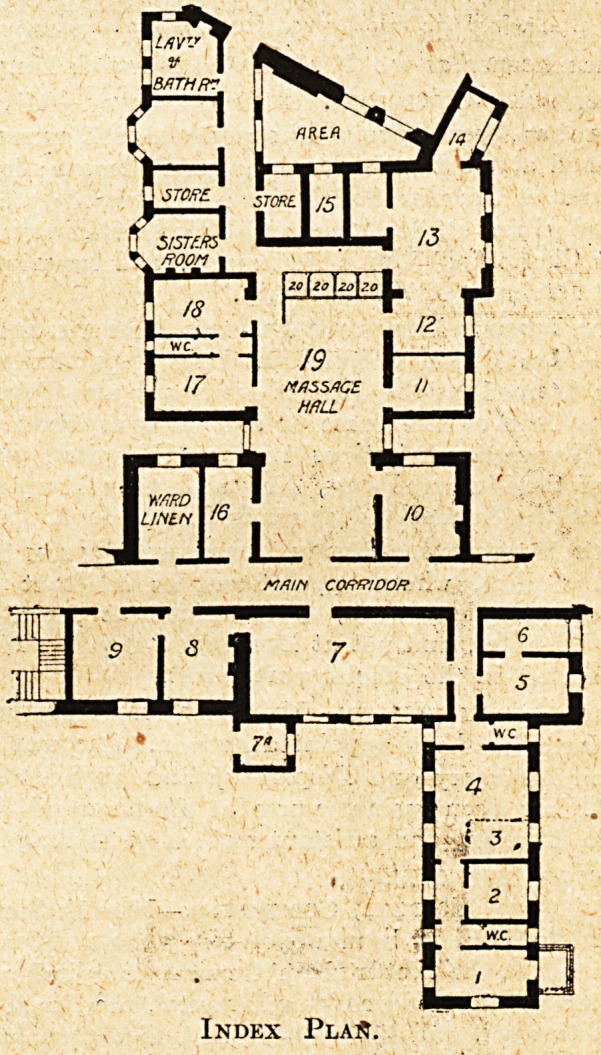# King Edward VII.'s Hospital, Cardiff

**Published:** 1918-04-13

**Authors:** 


### HOSPITAL ARCHITECTURE AND CONSTRUCTION.

### KING EDWARD VII.'S HOSPITAL, CARDIFF.

### X-Ray, Electrical, and Mechanical Hydro-Therapeutic Department for the Pre- and After-Treatment of Orthopædic Cases.

The electrical pavilion at this hospital has been in existence since 1905. It has been added to from time to time, and in the early part of 1916 Colonel Bruce Vaughan was so convinced that the treatment afforded by the French Government for the benefit of their wounded men should be afforded, if possible, to our own men at Cardiff that he persuaded the Board of Management very largely to increase the accommodation of the electrical department, which should include an extension of the massage department, so that the most up-to-date treatment by hydro- and electro-therapeutics and by Swedish remedial methods should be available at this hospital.

By these means it is not only hoped to hasten the recovery of the wounded and to fit them again for active service or their civil occupations, but also to give the same advantages to those who may become crippled in the course of their work in the various industries of the district.

All the rooms are well lighted, ventilated, and heated by radiators from the central system. The walls internally are lined with Minton's tiles glazed white as a wall filling with a dado of green tiles. The hall is open to the roof.

There are on an average 120 patients under treatment. These are sent by their various surgeons and physicians, and are then left under the care of the medical officer in charge of the department.

The department is well equipped, and steps are now being taken to provide other additional equipment, so as to add to its efficiency and in order to cope still more thoroughly and expeditiously with orthopædic cases. It is the intention of the board to place at once the equipment on a level, to some extent, with the great department at Hammersmith.

Attached to the department is a training-school for trained nurses in electricity, a school of massage recognised as a training-school for students in preparation for the examination of the Incorporated Society of Trained Masseuses; non-resident and a limited number of resident pupils are received for the terms commencing in May and November.

### THE PLAN EXAMINED.

A reference to the block plan (The Hospital, April 17, 1909) explains the difficulties of the situation. Further extension to the north-east is barred by the laundry block and mortuary. Taking into consideration the fact that modern orthopædic work is a new department, the development of which in the future can hardly be forecast, it would surely have been a wiser policy to have sacrificed the laundry and planned the new department on better lines, and with a careful eye to future extension.

Hence the impression that a study of this plan produces is that the building has been compressed into the smallest possible space, without due regard to light and air to it or to existing buildings. The position of the large ward, with the surrounding narrow areas and its badly planned sanitary annexe, cannot be regarded as satisfactory, and the very narrow area between the electric baths and the Shand Wing is the sort of arrangement one expects to find in a crowded block of offices in London rather than in a hospital in a city like Cardiff.

### THE DEPARTMENT'S STAFF AND EQUIPMENT.

The following list gives the staff, fittings, and equipment of the new department. The figures correspond with those on the index plan (see p. 39), and when read together the list thus shows the contents of each room.

X-Ray and Electrical Section.

*Staff.*—One doctor in charge, one sister, one staff nurse who is well trained, one probationer nurse.

*Equipment.*—1. Waiting-room. 2. Contains four-cell Schnee bath. 3. *X*-ray apparatus for treatment—*i.e.* switchboard, couch, and all accessories for treatment by *x*-rays. 4. One medical switch-table, also dynamo which supplies that room and the eye magnet in ophthalmic theatre with continuous current. 5. Sister's office, *x*-ray plates, stereoscope, desk, books, etc. 6. Dark room for

**KING EDWARD VII.'s HOSPITAL**—(*continued*).

developing photos, and containing a viewing box. 7. Universal $x$-ray couch, for radiograph, screening-stand with coil on top, trolley switch-table stand and shelf for $x$-ray tubes, viewing-box on switch-table. Table for diagnosing and locating bullets and foreign bodies. 7*a*. Dynamo-room, switches in room for transposing current to D.C. for eye magnet, etc. 8. High frequency (Gaiffe) apparatus with resonator, couch, and insulating platform, four-cell Schnee bath. 9. Two large porcelain arm-tanks for giving baths, sinusoidal, etc., modulation, instrument steriliser, and diathermy apparatus. (Average attendance for treatment daily, fifteen for skiagrams, screen examinations, muscle testing. Ordinary photographs, about twelve.) *For muscle-testing.*—One medical switch-table (combined galvanism and faradism), one constant battery with twenty-eight cells, portable, one Bristowe coil, two plurostats.

Mechano-Hydro and Therapeutic Section.

*Staff.*—One medical officer, one sister, trained nurse, and masseuse, one staff nurse, one masseur, one female attendant, four pupils.

Number attending, 107. Average number of patients treated daily, fifty-four (about twelve in the afternoon, the rest in the morning). Nearly every patient receives two forms of treatment, such as baths, massage, or electrical treatment and massage.

*Equipment.*—10. Medical officer's examination room. Two couches, one Bristowe coil, one multostat, one boom with saddle. 11. Whole-body agitation bath and couch. 12. Whirlpool bath for arm, whirlpool bath for leg. 13. One whole-body porcelain bath for sinusoidal current, with interrupter; one smaller porcelain bath for sinusoidal current, with interrupter (this bath is also fitted with hot and cold taps for fatty wounds); three couches. 14. Vichy douche, Scotch douche, and needle spray. 15. Sedative pool-bath. 16. Patients' waiting-room. 17. One whole-body semi-reclining radiant-heat bath, one couch. 18. One couch, two radiant-heat tables, one radiant-heat leg-bath. 19. One Sargent combined pulley machine with towing attachment, one stationary bicycle, three wrist instruments for flexion and extension, three wrist instruments for supination and pronation, one wrist instrument for lateral movement, one wrist roller, one shoulder instrument for rotation, one hip instrument for rotation, one ankle instrument for flexion and extension, one high plinth, one low plinth, one boom with saddle, one punch-ball, one

Index Plan.

walking-chair, twelve massage-stools, six massage-tables, one wall-bar, one blackboard with numbered holes and pegs for re-educational exercises, electrical apparatus, two pedestal vibrators, three Bristowe coils, two metronomes. 20. Dressing-boxes.

## Figures and Tables

**Figure f1:**
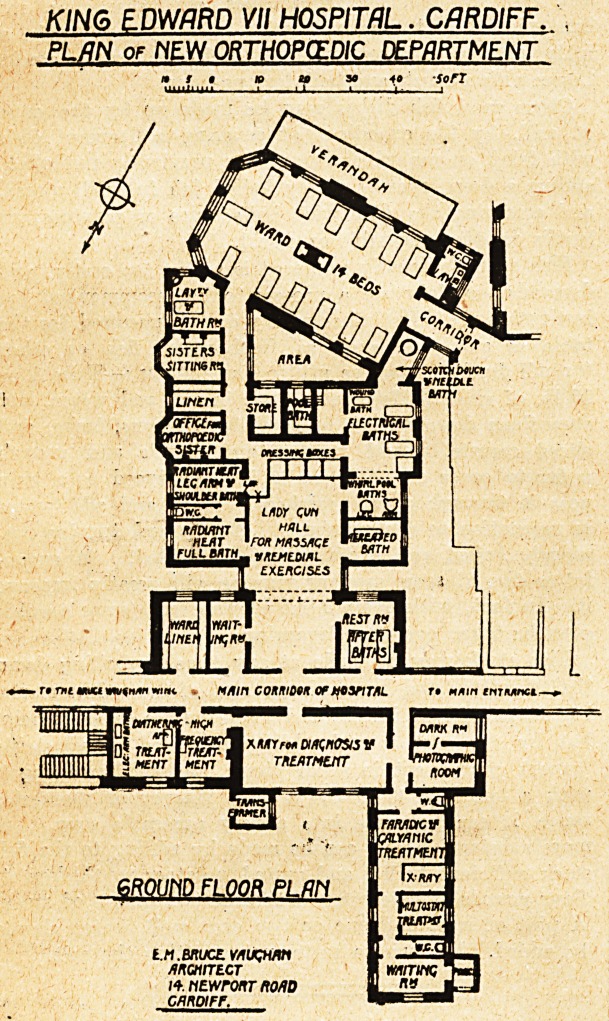


**Figure f2:**